# Supplementary material for: Research Progress on the Pharmacodynamic Mechanisms of Sini Powder against Depression from the Perspective of the Central Nervous System
Source: Medicina (Kaunas). 2023 Apr 10;59(4):741. doi: 10.3390/medicina59040741 (PMC10141708; doi:10.3390/medicina59040741)
Supplement: Supplementary file 1 [file medicina-59-00741-s001.zip › medicina-2285134-supplementary.pdf]

Supplementary Tables

Supplementary Table S1. Targets of top 3 MCODE clusters.

Cluster	Description	Targets
1	Apoptosis	CCND1, EP300, ALB, BCL2L1, CASP3, CASP9, HSP90AA1, MTOR, PTGS2, XIAP, SRC, TNF, ESR1, HRAS, AKT1, VEGFA, PGR, IGF1R, SIRT1, MAPK1, CDK4, MDM2, CDK2, CCNA2, ATM, TERT, PARP1, MCL1, CDK1, MMP2, MMP9, CXCL8, MAP2K1, CASP8
2	Cell cycle	ABCB1, NR3C1, KDR, ERBB2, EGFR, KIT, HNF4A, TOP1, HSP90AB1, ABL1, MAPK8, SLC2A1, AR, MET, FGF2, MAPK14, CCND2, PIK3CA, CDK6, APP, PRKDC, ERBB4, FYN, JAK2, HDAC1, PPARG, PPARA, GSK3B, TGFB1, ALK, MMP7, CASP1, MMP3, MMP1, NOS3, SERPINE1, PLG, IL2, ESR2, RPS6KB1, CDC25A, EIF2AK3, CDC25C, PTK2, CHUK, CHEK1, FGFR1, IKBKB, RET, CHEK2, PLK1
3	PI3K-Akt signaling pathway	AGTR1, ABCG2, HPGDS, SMARCA4, FLT3, RARA, PDGFRB, CREBBP, MAPK11, LCK, JAK1, ATR, RAC1, PTPN1, PRKCD, LYN, PIK3R1, NTRK1, PDGFRA, MMP14, ARG1, TLR4, PRKCA, MMP13, PKM, HSPB1, BTK, HDAC5, CCR2, INSR, HDAC6, PIK3CG, RAF1, NOX4, NCOR1, CSK, LGALS3, HDAC4, PIK3CB, DAC3, NOS2, TLR9, PIK3CD, PRKCZ, KDM1A, CCNE2, HDAC2, FGF1, FLT4, ROCK1, SYK, FGFR2, PCNA, FEN1, FGFR3
